# Bronchodilator reversibility testing in morbidly obese non-smokers: a comparative study– few concerns

**DOI:** 10.1186/s12890-024-02951-9

**Published:** 2024-03-15

**Authors:** Divya Balan, Manish R Shetty, Manu K Mohan

**Affiliations:** Department of Respiratory medicine, Kasturba Medical College Manipal, Manipal Academy of Higher Education, Manipal, Karnataka India

## Abstract

This is a letter in response to an article by Ahmed et al., which concluded that in comparison to salbutamol, Fluticasone/salmeterol combination increases FEV_1_, FEV_1_% of predicted, and FEV_1_/FVC ratio, however it did not offer novel insights, as both agents met the 12%- and 200-mL reversibility benchmarks and Concerns about incorporating a combination medication that includes an inhaled corticosteroid, inhaled corticosteroids are not typically associated with bronchodilation.

To the editor,

We read the article, titled ‘Bronchodilator reversibility testing in morbidly obese non-smokers: comparing the effectiveness of fluticasone/salmeterol to salbutamol bronchodilator’, with great interest [1]. We appreciate the authors for their well-written content. We have a few concerns.

The aim of this study was to assess and compare the efficacy of fluticasone/salmeterol to salbutamol as agents for early reversibility testing in population with varied body mass indices. However, I would like to express my concern about incorporating a combination medication that includes an inhaled corticosteroid, inhaled corticosteroids are not typically associated with bronchodilation. Inhalation corticosteroids (ICS) exhibit limited bronchodilator effects, especially over a short duration of time. The primary function of ICS is to control inflammation in the airways, making them more effective as controllers of conditions like asthma. These medications suppress inflammation, primarily by deactivating multiple activated pathways associated with airway inflammation [2, 3]. Salmeterol is a long-acting bronchodilator commonly used to manage asthma and chronic obstructive pulmonary disease (COPD). It has a slower onset of action compared to short-acting bronchodilators like salbutamol. The onset of action for salmeterol typically occurs within 20 min, which is longer than short-acting bronchodilators like salbutamol which work within 5–15 min [4]. The peak action of salmeterol, where it provides the maximum bronchodilation, usually occurs after approximately 1 to 2 h of administration. This extended duration of action is why it is considered a long-acting bronchodilator and is typically used for maintenance therapy rather than for rapid relief of acute symptoms. In summary, salmeterol’s onset of action is around 20 min, making it unsuitable for rapid relief of acute bronchospasms, and its peak action occurs within 1 to 2 h after administration, providing extended bronchodilation for the management of chronic respiratory conditions [4].

Moreover, the study defines reversibility as positive in patients whose FEV1 exhibits a minimum of a 12% improvement from the baseline value and a significant increase of at least ≥ 200 mL following bronchodilator administration. Interestingly, the results of this study reveal that all three study groups display a notable change in FEV1% predicted, exceeding 15.5% when administered salbutamol as the bronchodilator. Notably, the combination drug yields superior outcomes, particularly in obese patients, with an improvement of approximately 20%.

However, it’s important to note that this finding doesn’t introduce any novel insights into the existing clinical guidelines which define bronchodilator reversibility. This is because the required level of reversibility is set at only 12%, and salbutamol and salmeterol/fluticasone effectively achieve this benchmark. This shows that the obese patients had reversible airway obstruction.

Hence, we prefer to disagree with the recommendation that salmeterol/fluticasone combination as preferred agent for reversibility testing instead of salbutamol alone in obese subjects, due to above mentioned reasons.
